# Case Report: Intensive Inpatient Neurorehabilitation Achieves Sustained Real-World Benefits in Severe Alcohol-Related Wernicke-Korsakoff Syndrome: A Case Study With 7-Years Follow-Up

**DOI:** 10.3389/fpsyg.2021.693920

**Published:** 2021-06-16

**Authors:** Mareike Schrader, Stephan Bamborschke, Ute Lenk, Annette Sterr

**Affiliations:** ^1^P.A.N. Zentrum für Post-Akute Neurorehabilitation, Berlin, Germany; ^2^School of Psychology, University of Surrey, Guildford, United Kingdom

**Keywords:** Wernicke-Korsakoff syndrome, cognitive deficit, societal participation, tobacco alcohol optic neuropathy, case study

## Abstract

About 85% of survivors of acute Wernicke's Encephalopathy (WE), a frequent and serious consequence of thiamine deficiency and alcohol misuse, sustain chronic neurocognitive deficits also known as chronic Wernicke-Korsakoff syndrome (WKS). If alcoholism is combined with smoking, tobacco alcohol optic neuropathy (TAON) may occur which leads to visual impairment. In contrast to WKS, TAON may be treated successfully by early vitamin substitution and detoxification. Little research has been conducted on WKS longterm outcomes. Existing literature suggests poor prognosis. Symptoms remaining beyond the acute treatment with thiamine are thought to be irreversible. Whether neurorehabilitation may be an effective route to help recovery of those persistent symptoms is an open question. At our neurorehabilitation center, which specializes in the treatment of severe chronic deficits after brain injury, the opportunity arose to treat a 35 year old male with WKS, and to conduct follow-up assessments 3- and 7-years post discharge, respectively. Initially MK was admitted to emergency care with suspected postconcussive syndrome, alcohol-related thiamine deficiency, and TAON. Thiamin, cobalamin, and folate substituion improved TAON but major cognitive deficits remained. When admitted to our center 4 months later, he was fully reliant on care staff for all activities of daily living (ADL). Through intensive neurocognive training and psychological treatment he improved gradually and, after 26 months, was well enough to be discharged into the community and pursue work in a sheltered setting. Neuropsychological tests, as well as patient reports obtained at the follow-ups showed that the benefits apparent at discharge had been sustained, and for some scores, improved further. This was particularly evident in the Rey-Osterrieth Complex Figure Test which improved from percentage ranges <1 for immediate recognition and recall at discharge to rank 16 for immediate recognition and rank 5 for recall at the 7-year follow-up. This case study illustrates the immense benefits neurorehabilitation can have for WKS induced by alcohol misuse. It further demonstrates how skills and strategies, learned in the inpatient setting, translate into living well and independently, and how the latter promotes further improvement long after discharge.

## Introduction

About 85% of survivors of acute Wernicke's encephalopathy (WE), a frequent consequence of thiamine deficiency and alcohol misuse, sustain chronic neurocognitive deficits also known as chronic Wernicke-Korsakoff syndrome (WKS). These deficits comprise antero- and retro-grade amnesia, executive dysfunction, visuospatial deficits, ophthalmoplegia, and ataxia (Arts et al., 2017). If alcoholism is combined with heavy smoking, tobacco alcohol optic neuropathy (TAON) may occur which leads to visual impairment caused by dyschromatopsia and bilateral central scotoma (Chiotoroiu et al., 2014; Roda et al., 2020). Tobacco alcohol optic neuropathy and Wernicke's encephalopathy, if diagnosed early, may be successfully treated in addition to thiamine substitution by application of cyanocobalamin (Vitamin B12), folic acid, and cessation of smoking and drinking alcohol (Thomson et al., 2012; Day et al., 2013; Zhu et al., 2019).

The neurocognitive deficits characteristic for WKS are very debilitating to the individual and detrimental to wellbeing, quality of life, and societal participation. Wernicke-Korsakoff syndrome symptoms further aggravate the huge challenge of effective interventions for alcohol misuse. This is a critical aspect since treatment for alcohol addiction is notoriously difficult with relapse rates after a period of abstince estimated to range from 68 to 85% (Batra et al., 2016; Zhu et al., 2019).

In Germany, just like many other European countries, the care pathway for WKS comprises the acute intervention with thiamine followed by referral to services providing treatment for alcohol use disorder (AUD). These services, however, do not address the neurocognitive deficits of WKS in any specific way (Svanberg and Evans, 2013), even though it is estimated that 50–80% of patients presenting to alcohol treatment services show signes of cognitive impairments (Bates et al., 2002). Thus, the argument has been made that WKS, albeit being caused by malnutrition and vitamin deficiency due to repeated alcohol intoxication, is essentially a disorder of the brain that requires neurorehabilitation (Bates et al., 2013).

To the best of our knowledge, only two case reports have addressed this issue (Monteiro et al., 2011; Genç et al., 2018), but none of them covered long-term follow-ups. In detail, one case study of a 48 year WKS patient with a history of heavy drinking, reported outcomes of inpatient neurorehabilitation (Genç et al., 2018). The second study reported the observations from a 42 year WKS patient treated in an ambulant neurorehabilitation setting (Monteiro et al., 2011). Other studies on cognition and WKS are concerned with the effect of early pharmacological intervention (Caballeria et al., 2020).

The case study presented in the present paper concerns a 35 year old male (MK) with severe alcohol-induced WKS and TAON who received 26 months of intensive inpatient neurorehabilitation at the Zentrum für Post-Akute Neurorehabilitation (P.A.N., Berlin Germany), and was followed up 3- and 7-years post discharge. MK has given written informed consent to the collection and publication of this data.

## Detailed Case History

**Figure 1:**
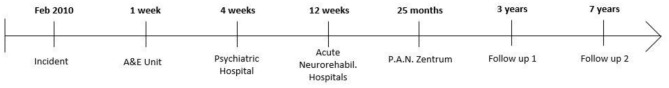
Timeline of events illustrates the journey of his incident to 7-year follow-up.

The time of care pathway is illustrated in Figure 1. MK had a long history of severe and debilitating alcohol addiction. In February 2010 he was found lying in a street after what appeared to be some form of accident under the influence of alcohol. He was taken to the Accident and Emergency Unit (A&E) of a general hospital in Germany with suspected postconcussional syndrome (ICD-10, F07.2). After 1 week he was transferred to a psychiatric hospital, where he presented with acute amnestic syndrome, impaired vision with dyschromatopsia and bilateral central scotoma, spatial disorientation, and unstable gait due to polyneuropathy; he used walking aids and needed help with all activities of daily living (ADL). At that point he was also diagnosed with WE and TAON and treated accordingly. When admitted to the psychiatric hospital MK was abstinent, probably for a week or so, as it is unlikely that he managed to obtain alcohol or tobacco while at A&E. However, this is not documented explicitly in the case notes available to us. Magnetic resonance brain scans (MRI), taken at A&E and the psychiatric hospital, showed moderate atrophy of the frontal cortex and the cerebellum but no other abnormalities, signs of lesions, or bleeding.

Four weeks after the incident, MK was transferred from the psychiatric hospital to two acute neurorehabilitation hospitals where he was treated consecutively for 12 weeks in total. From there he was transferred to P.A.N., because no progress toward recovery had been achieved.

## Presentation at Admission to P.A.N. (2010)

The discharge letter describes MK as disoriented, needing help and/or guidance with all basic ADL, and an inability to adhere to any form of routine without constant reminders. This picture was confirmed by the entry assessment at P.A.N., which additionally noted, gait ataxia, personality changes, temporal and spatial disorientation, and a lack of awareness. Vision was mainly recovered but the patient was anxious because of disorientation and feared to become blind again. The goals MK stated at admission comprised living independently ideally in his own flat, pursuing work, and staying abstinent from alcohol and tabacco.

Neuropsychologically MK presented with retrograde amnesia of approximately 2 years, near complete anterograde amnesia, and attention deficits. He also suffered from severe anxiety and volatile mood. Working memory, short-term memory, and procedural long-term memory seemed preserved. His biographical memory up to 2 years pre-incident also appeared to be fully intact.

Formal neuropsychological test results are summarized in Table 1, column 2. The Verbaler Lern- und Merkfähigkeitstest (VLMT) (Helmstaedter et al., 2001) revealed substantive deficits in learning, encoding, recognition, and free recall, but good short term- and working memory. Poor performance was observed in the Test of Attention Performance (TAP) (Zimmermann and Fimm, 2002) subtests alertness, divided attention, and Go-Nogo, suggesting a general reduction in processing speed and limited attentional capacity with preserved response inhibition. Performance in the trail making test (TMT) (Reitan and Tarshes, 1959; Tombaugh, 2004) was good with 31 s for part A and 74 s for part B, respectively. IQ was normal (108; HAWIE-R) (Tewes, 1991). The Tower of Hanoi was completed with ease, suggesting good executive function (Simon, 1975). His cognitive abilities were such that he was not able to complete the Berliner Amnesie Test (BAT) (Metzler et al., 2010) or the Rey figure (Meyers and Meyers, 1995).

**Table 1:** Summary of the neuropsychological tests conducted at P.A.N. admission (2010), P.A.N. discharge (2012), 3- (2015), and 7-year follow-up (2019) in the community.

	**2010**	**2012**	**2015**	**2019**
**BAT** (z-scores; PR)				
Amnesia score			−12; PR = 0	−1.5; PR = 7
Verbal score	n/a	n/a	−7; PR = 0	−3; PR = 0
Figural score			−9.4; PR = 0	1.5; PR = 93
Amnestic syndrom			−12.9; PR = 0	−1.2; PR = 12
**TMT A/B** (s)	31s/74s	–/98s	–	28s/94s
**Rey Figure** (raw score; PR)				
Immediate	n/a	11.5; PR = 1	11; PR = 1	16.5; PR = 16
Recall/delayed recall		4; PR <1	8; PR <1	14.5; PR = 5
**TAP**				
Alertness (key value; PR)	−0.022; PR = 21		0.011; PR = 34	−0.046; PR = 16
- uncued (s; PR)	315s; PR = 4		274s; PR = 12	238s; PR = 34
- cued (s; PR)	322s; PR = 5		271s; PR = 12	249s; PR = 21
Working memories (no.; PR)		_		
- Omissions	n/a		0; PR = 88	0; PR = 92
- Errors	n/a		5; PR = 14	3; PR = 38
Divided Attention (no.; PR)				
- Omissions	4; PR = 8		0; PR = 79	0; PR = 82
- Errors	4; PR = 8		0; PR = 76	0; PR = 79
Go-Nogo (no.; PR)				
- Errors	1; PR = 38		0; PR > 42	0; PR > 42
- Med response time (s; PR)	668s; PR = 2		508s; PR = 62	491s; PR = 79
**VMLT** (raw score; PR)				
- Encoding	6; PR = 30–45		9; PR = 85	8; PR = 75–80
- Learning	29; PR ≤ 5	_	41; PR = 10	41; PR = 10
- Consolidation	5; PR ≤ 5		6; PR ≤ 5	0; PR = 70–85
- Free recall	0; PR ≤ 5		2; PR ≤ 5	9; PR = 15–20
- Recognition	0; PR ≤ 5		1; PR ≤ 5	8; PR = 15–20

*n/a, patient lacks cognitive ability to perform the test; PR, percentil rank; s, seconds*.

## Treatment at P.A.N.

### General Characteristics

**Figure 2:**
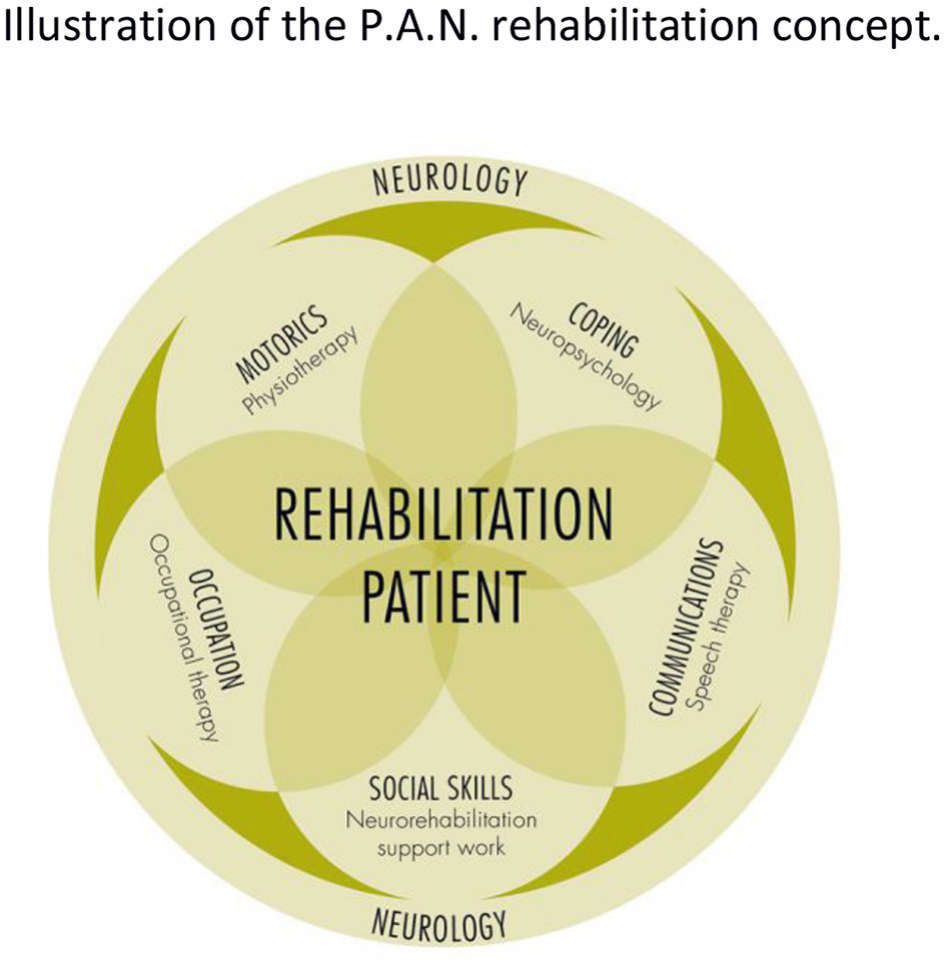
Illustration of the P.A.N. rehabilitation concept. Hallmark of this concept is a holistic approach with interdisciplinary support of rehabilitation, where all therapeutic intentions are tied into 24/7 program of learning to live independently. Patients live in groups of 14 in a home-like setting. Within these groups level of support is titrated to individual needs and hence allows for “round the clock” practice and the continous adjustment of goals. Going through neurorehabilitation while living in groups further supports communication and practice of social skills.

P.A.N. uses a holistic psychosocial approach where therapeutic interventions are tied into a 24/7 program of learning to live independently (see Figure 2). Thus, patients live in groups of 14 in a home-like situation on the P.A.N. campus. This living arrangement affords individual titration of ADL support needs on a daily basis and allows for “round the clock” practice of the skills and strategies learned in treatment sessions with e.g., physio-, occupational-, or language therapists.

### Treatment of MK

Initially MK's most pressing difficulties concerned the inability to compensate for his severe memory deficits and/or use of memory aids and lack of insight which led to severe disorientated in space and time. Moreover, he was agitated, had volatile mood swings and high levels of anxiety. The experience of visual loss in the early phase of the disease had been emotionally so important, that it was still present in his memory. During the first months MK was permanently scared to get a relapse. When he learned that becoming blind could only be avoided by strict alcohol and tobacco abstinence this became a very strong and reliable motivation to change his lifestyle.

Through a wide range of therapeutic activities he was gradually taught to rely on calendars and checklists as memory aids. A fine-grained repetitive approach, where tasks and instructions were broken down into small (sometimes minute) steps, was adopted for everything from basic hygiene to complex ADLs, activity planning, and social interaction. A similar strategy was used to treat the disorientation: he first learned to use notes and maps as orientation aids to find rooms within the building and places on the P.A.N. campus. He subsequently learned to use google maps to navigate the vicinity of the P.A.N. safely and reliably. He also learned to combine the use of external aids such as structured instructions, memory aids, and maps to plan activities in advance.

A good example to illustrate the approach is when MK learned to use the copy machine. Several times a day he practiced to use it with notes and memory aids provided by the therapist. At the beginning of the process he would not remember that he'd used the copy machine on the day before or that he had notes to refer to. But gradually, the combination of prompts, practice, and persistence helped him to learn to consult his notes which then enabled him to use the machine. This was a very slow process with improvements so small that they could easily have been dismissed as lack of progress. However, over time the reliance on notes, checklists, and memory aids became a coping strategy MK learned to rely on. Whenever he struggled to achieve his goal or “got stuck,” it became an “automatic behavior.” Moreover, initial successes achieved through the effective use of memory aids became a driver for empowerment. The gradual achievements promoted insight into his deficits and, in turn, led to greater acceptance of external memory aids. The latter was rewarding and increased confidence. Together these psychological impacts fuelled a self-perpetuating motivational cycle of goal setting and practice that allowed progression from using the copy machine to using notes for handling emails, cooking, or sorting laundry. Eventually he progressed to drafting his own memory aids.

The progress evident in the above account is impressive. However, this should not disguise the fact that this was a challenging journey with many setbacks, e.g., MK would be given the instructions to find a therapy room but forgot that he had the instructions; he was given prompts but forgot to use them etc. These frustrations were carefully managed through psychological support; the setbacks were overcome by practice, practice and more practice so that eventually the strategy “check your pockets, look for notes” became so embedded in his behaviors that it became the automatic response when faced with challenges. While most of the treatment in relation to the above was conducted on a one-to-one basis, MK also participated in group activities, such as cooking, computer training, photography, and board games. These activities were structured for him so that the strategies learned in one-to-one sessions could be applied in group settings. For example, the cooking group would plan a meal for the coming week. It was his task to write the shopping list and then run some of the errands a few days later.

MK was very keen to get well enough to pursue some form of employment. Specific steps toward this goal were taken about 15 months into his stay. P.A.N. is equipped with training workshops for wood craft and textiles. Here, patients work with occupational therapists using diaries and daily goal setting. Thus, in the last half hour of the working day, patients complete a diary with what they have done and what they intend to do next. These notes are then consulted the next day to determine the work plan for the session ahead. Through this iterative process of reflection and planning patients learn to use their notes effectively and gradually gain a more independent way of working. In these facilities MK completed a trial of part-time work (3 h per day, 4 days a week) with great success. He then secured a 2-week industrial internship off site. Eventually he secured employment in sheltered workshop (Behindertenwerkstätten) in the community.

MK's biggest and most challenging goal comprised his wish to live independent in his own flat. Through the living arrangements and therapeutic approach practiced at P.A.N., work toward this goal was embedded in all activities from day one. Training of the skills to master everyday life was performed within the community and included activites such as using the bus, going to the shops, and purchasing goods. This training thereby not only comprised practice sessions but also therapy time for reflection. These reflection sessions formed an integral part in managing MK's high levels of anxiety and, critically, the danger of breaking his abstinence. The risk for the latter rose with increasing levels of independence, since the ability to go shopping without help also meant opportunity to purchase alcohol and tobacco. This risk was managed through behavioral contracts drawn up between patient and therapist before e.g., an independent trip to the shops. Moreover, MK's fear of relapse and the consequential return of TAON presented a strong motivator for maintaining abstinent.

## Discharge

After 26 months at P.A.N. MK's care needs had reduced substantially from 154 points (level 5) to 111 points (level 3) out of a scale 1–5 HMB-W method^1^ so that he could be the discharged into an apartment, shared with three others, located in a sheltered housing complex with low levels of support.

## Three- and Seven-Year Follow-Up

The outcome of the neuropsychological assessments are presented in Table 1 columns 4 and 5. The categorical classification of percentile ranks (PR) values are presented in Table 2. In essence all tests demonstrated that MK's cognition was stable or had improved further. Data from the TAP indicated progressive improvement in attentional performance that continued to 2019, with speed of processing being close to norm level, and performance increases from PR = 12 in 2015 to PR = 21 and 34 for cued and uncued attention, respectively. Working memory improved from a subnormal score in 2015 to the normal range in 2019. Dual-task performance (divided attention) was well below the norm in 2010 but reached normal levels in 2015 and excellent levels in 2019. The parameters for response selection and effective inhibition (TAP Go-Nogo subtest) revealed good performance in 2010, and fell at the upper end of the norm distribution in 2015 and 2019. Taken together the follow-up assessment of the TAP demonstrates a good restoration of attentional deficits.

**Table 2:** Categorical classification of percentile rank (PR) values according to the German Guidelines (www.neuropsy.ch; category descriptors translated by the authors).

**Percentil rank**	**Classification**
>98–100	Very superior
≥95– ≤ 98	Superior
≥84– <95	High average
≥16– ≤ 84	Average
>5– <16	Below average
≥2– ≤ 5	Very low
0– <2	Extremely low

The learning deficit evident in the VLMT of 2010 was slightly improved in 2015 and remained stable in 2019. However, the free recall and recognition subtests indicate a slightly better ability to retain learned material.

The BAT was only acquired in 2015 and 2019. In 2015 the BAT amnesia syndrome score was very poor in all subtests (PR = 0). In 2019 however BAT scores improved remarkably, with the biggest improvement observed for the figural score (PR = 93).

**Figure 3:**
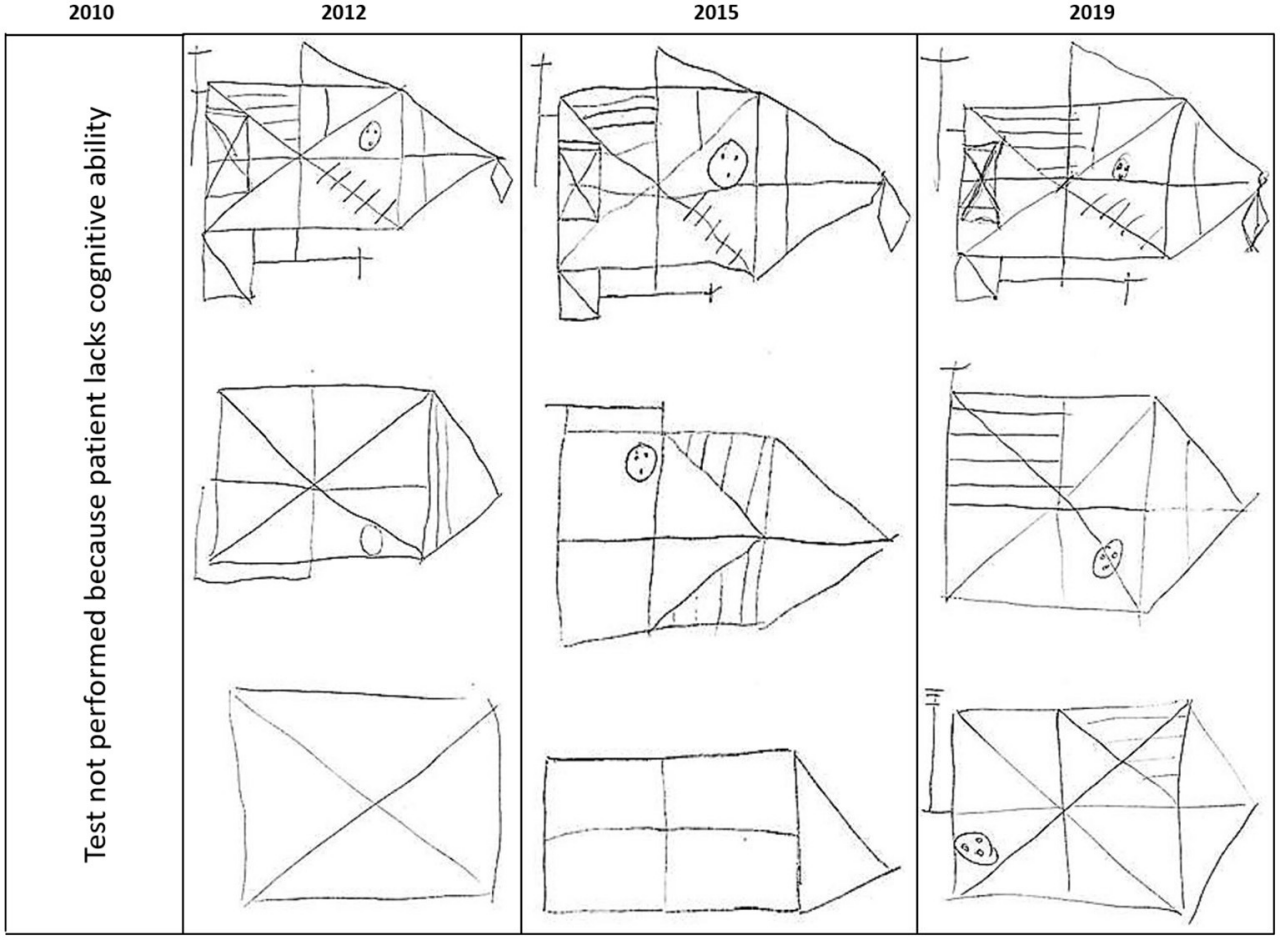
Performance on Rey figure.

The Rey figure is the only test conducted at the 2012 discharge as well as the two follow-up sessions. At admission MK's cognitive deficits were to severe to do this test; at discharge he was able to complete the test but performance was very poor (PR <1). In 2015 this performance had improved, and was best in 2019 (PR = 16 for recognition; PR = 5 for recall at the 7-year follow-up). The test results (drawings) are presented in Figure 3.

## Assessor Observations and Patient Perspective

For the 3-year follow-up, MK came to the clinic independently. He recognized some of the staff and fondly commented on his long stay at P.A.N. and how it had changed his life. By now, he had moved to his own apartment in the community where he lived with very minimal support. The clinical interview showed he was doing well. He was able to walk without aids and had largely recovered his vision. He reported to be still abstinent from alcohol and tobacco, and that he continued to live in his own flat with minimal assistance. He further reported that soon after discharge from P.A.N. he had started to work for the industrial employer, where he originally completed his internship. To get to work he completed a long and relatively complicated journey on public transport. MK's own judgement was that he was doing well, both privately and at work. Correspondence with the work supervisor confirmed that MK's work performance was very good. In addition it was noted that he required extra support for some tasks at work.

The 7-year follow-up was done at home. MK again recognized his main therapist and remembered his time at the clinic well and fondly. He was still abstinent from alcohol and tabacco, and continued to work in the same role. In his private life, he did all the shopping by himself, but had some help with cleaning and with organizing activities that were out of the ordinary, e.g., a visit to a new doctor. He lovingly and reliably cared for his pet parrott.

Assessing MK at home gave further insights into how he managed his daily life. Thus, he made extensive and ritualized use of external memory aids, such as stickies with prompts and reminders notes in various places around his flat. He also kept diaries to keep notes about himself/his own history, e.g., stories from the past his mum had told him. Socially he had weekly contact with his family but otherwise struggled to find friends. Organized through an assisted living service, he had joined a cooking group to help with social isolation. He had tried to go on holiday by himself once, but was to disorientated to manage and aborted the trip. According to his own judgement he was happy and content but very much wished to find a girlfriend.

## Discussion

To date, only two published papers have focussed on the effects of neurorehabilitation in chronic WKS (Monteiro et al., 2011; Genç et al., 2018). Monteiro et al.'s case study (Monteiro et al., 2011) found good outcomes following 25 weeks of ambulant neuropsychological training in a 42 year old female with a 15-year history of worsening tobacco and alcohol misuse. In contrast to our study, the person was well enough to live at home throughout the 25 weeks of rehabilitation, i.e., had much lower needs. The authors further noted the critical role of family (cohabitants) in the daily transition from the therapy to the home setting with family member effectively acting as co-therapists. Genç et al.'s case study (Genç et al., 2018) on the other hand reported positive outcomes of 8 weeks of inpatient physio- and occupational-therapy in a 48 year old male with a 30 year history of heavy drinking. The present study not only strenghtens the idea that neurorehabilitation may be beneficial to patients with WKS in a chronic state, but extend these observations significantly. Specifically, our study demonstrates sustained benefits of intensive neuropsychological rehabilitation from serious alcohol-induced neurocognitive deficits, including WKS and TAON, for 7-years post intervention. This conclusion, however, has to be contextualized by the limitations arising from the case study design in general, and the absence a comprehensive neuropsychological profile at the point of discharge.

Prior to P.A.N. admission, life in a nursing home for alcoholics was the only prospect for MK. But through prolonged neurorehabilitation, he was able to recover skills and functions and to learn strategies that allowed him to live well and independently, to pursue work, and to remain abstinent. Critically, this case study further shows that the benefits obtained through inpatient neurorehabilitation were not only maintained after discharge, but further neurocognitive improvements took place thereafter. This is remarkable for two reasons. First, alcohol-related brain damage is thought to be permanent and hence not reversible. Secondly, alcohol misuse is thought to accelerate age-induced cognitive decline and one might therefore predict a natural decline over time, i.e., lower cognitive performance at 7-year follow-up. But the opposite is the case. Our paper therefore goes beyond the study by Bruijnen et al. (2021), who demonstrated cognitive recovery from alcohol-induced deficits after 6 weeks of abstinence. Moreover, our results from the Rey figure indicates that MK's cognition improved post discharge, i.e., without specific neurocognitive interventions. We postulate that these longterm effects are largely driven by the positive effect independent living has on the preservation of cognitive function. Thus, studies in healthy persons have identified an active live style with good social participation as effective countermeasures for cognitive decline (Ong et al., 2016). One might argue that MKs continued improvement after discharge from rehabilitation could, at least in part, be explained by the fact that he was able to live an active and self-determined life.

WKS is often seen as a memory disorder, but actually the condition presents with a mix of cognitive and behavioral symptoms comprising memory and executive dysfunction, apathy and disorders of affect, emotion perception, and social cognition [see (Arts et al., 2017) for review]. The case of MK confirms WKS as a complex syndrome with cognitive, behavioral, and emotional symptoms. It further shows how these complex needs can be addressed in the rehabilitation process and treated successfully. Importantly MK's treatment at P.A.N. combined neuropsychological and psychological treatment to help him learn how to cope with his cognitive deficits and also his emotional problems. We speculate that this was central to adopting and maintaining an alcohol free life, which formed the foundation for successful neurorehabilitation and subsequent return to the community. Taken together, these results lend strong support to the notion that alcohol-related neurocognitive deficits can be treated through neurorehabilitation. In addition, the neuropsychological test profile combined with the observations summarized in section Treatment at P.A.N., suggests that errorless learning might be particularly important in the treatment of WKS patients. Moreover, another key to the successful treatment of MK was the acquisition and application of strategies. These benefits were delivered mainly through one to one treatment. In the future this might be achieved more cost effectively through new technologies. For example, Lloyd et al. (2019) recently demonstrated how smartwatches can aid prospective memory in patients with WKS.

Another remarkable observation in this case concerns the early diagnosis and successful treatment of TAON. According to MKs own judgement, this experience of TAON was emotionally so important, that the fear of a relapse was the main motivation of long lasting abstinence to alcohol and tobacco.

## Data Availability Statement

The original contributions presented in the study are included in the article/Supplementary Material, further inquiries can be directed to the corresponding author/s.

## Ethics Statement

Written informed consent was obtained from the individual for the publication of any potentially identifiable images or data included in this article.

## Author Contributions

MS and AS: writing paper, analysis, and interpretation. UL: data collection. SB: interpretation and study planning. All authors contributed to the article and approved the submitted version.

## Conflict of Interest

The authors declare that the research was conducted in the absence of any commercial or financial relationships that could be construed as a potential conflict of interest.

## Footnotes

^1^ICF-aligned assessment tool used by the federal health- and social-services of Berlin to assess support needs in persons with disabilities.
